# Challenges in horizontal model integration

**DOI:** 10.1186/s12918-016-0266-3

**Published:** 2016-03-11

**Authors:** Katrin Kolczyk, Carsten Conradi

**Affiliations:** Max-Planck-Institute Dynamics of Complex Technical Systems, Sandtorstr. 1, 39106, Magdeburg, Germany

**Keywords:** Horizontal model integration, Model merging, Model reparameterization, Integration workflow

## Abstract

**Background:**

Systems Biology has motivated dynamic models of important intracellular processes at the pathway level, for example, in signal transduction and cell cycle control. To answer important biomedical questions, however, one has to go beyond the study of isolated pathways towards the joint study of interacting signaling pathways or the joint study of signal transduction and cell cycle control. Thereby the reuse of established models is preferable, as it will generally reduce the modeling effort and increase the acceptance of the combined model in the field.

**Results:**

Obtaining a combined model can be challenging, especially if the submodels are large and/or come from different working groups (as is generally the case, when models stored in established repositories are used). To support this task, we describe a semi-automatic workflow based on established software tools. In particular, two frequent challenges are described: identification of the overlap and subsequent (re)parameterization of the integrated model.

**Conclusions:**

The reparameterization step is crucial, if the goal is to obtain a model that can reproduce the data explained by the individual models. For demonstration purposes we apply our workflow to integrate two signaling pathways (EGF and NGF) from the BioModels Database.

**Electronic supplementary material:**

The online version of this article (doi:10.1186/s12918-016-0266-3) contains supplementary material, which is available to authorized users.

## Background

For studying biological processes at the pathway level plenty of mathematical models have been developed. Answering new and even more complex biomedical questions requires models of complete cells, organs or even organisms. An arguably very efficient approach to obtain such models is to combine or integrate existing models. An ideal starting point are the continuously growing model databases, for example the BioModels Database [1], the CellML Model Repository [2] or the JWS Online - Model Database [3]. Thus model integration may potentially speed up the systems biology cycle of modeling and experimentation by re-using the data that was explained by the individual models. Moreover, by combining existing data and models one may obtain an integrated model of enhanced predictive power.

In general, model integration can be subdivided into vertical integration (i.e. integration of models across formalisms and scales) and horizontal integration (i.e. integration of models which use the same formalism and scale). While much effort has been put into vertical integration [4–6] we want to emphasize that horizontal integration is an important task that deserves special attention. Thereby we concentrate on the integration of two kinetic ODE models A and B and address two challenges. The first challenge arises when the models are merged: identical model elements (e.g. chemical reactions and species) have to be identified. We propose to use a merged model that contains every element only once. For every reaction occurring in both models one has therefore to decide which parameter values to choose, either those used in model A or those used in model B. Similarly, for every species occurring in both models one has to decide which initial values to choose. Clearly this choice of parameter values and initial values affects the simulation results of the integrated model and hence the ability of the integrated model to explain the experimental data that was used to parametrize models A and B. Here the second challenge arises: to obtain a parameterization of the integrated model. In our point of view model integration is only successful, if the integrated model is consistent with the experimental data used to parametrize models A and B. A precise definition is given later on.

To address the first challenge we present a naming scheme that simplifies the identification of identical model elements. This naming scheme was originally developed in the context of the Virtual Liver network, but is applicable to most ODE models arising in systems biology. With respect to the second challenge we first note that the naive way to obtain a consistent model, namely discarding all parameter values and parameter re-fitting, is hampered by high computational cost and limited availability of experimental data. Hence we suggest to reuse the parameterization of the original models to a large extent. To this end we discuss ideas to retain many parameter values while adapting only (very) few. Of course, such a model has to be validated, both theoretically and experimentally. It is an ideal staring point for numerical studies like stability and sensitivity analysis that can be performed at almost no additional cost.

To facilitate the complete integration process we propose a semi-automatic integration workflow. Thereby we distinguish between ‘structural integration’, the merging of model elements (networks) and ‘behavioral integration’, the adaption of parameter values to obtain an integrated model that is able to explain experimental data.

The term ‘integration’ will be used throughout this document to describe the whole process of fusioning the existing models to obtain a simulatable model which is able to explain experimental data. In the literature also the terms ‘merging’, ‘composition’, ‘combination’ or ‘aggregation’ can be found to describe this process [7–9]. We will make use of the term ‘merging’ in the context of combining the networks.

Before turning to challenges and workflow we discuss existing standards and software which can support model integration in the following two subsections. In the subsequent section ‘Results and discussion’ we introduce the model integration workflow and discuss challenges and potential solutions in structural and behavioral model integration. As a proof of principle this workflow is applied to the integration of two signaling networks originally described in [10]. The details can be found in the final ‘Methods’ section of this paper.

### Existing standards support model merging

Merging of models from smaller submodels is a common practice in working groups. There models are often merged by hand in a straightforward way because mostly the same software tools and formats are used. One important task in model integration is to find the model overlap. The overlap of two models comprises all model elements (reactions, species, parameters, compartments) which are contained in both original models. Within working groups the semantic meaning of a model and its elements is known or can be communicated on a short way. Hence, the model overlap can often be found easily. Whereas, finding the model overlap of models which originate from different groups and integrating such models in various combinations can be challenging.

Usually, kinetic models in systems biology contain all mathematical information which is needed for simulation but lack semantic information needed to find elements which describe the same biological component or reaction. To discover identical model elements in different models the assignment of information to the model and the application of common modeling standards and guidelines is required. This is also an important prerequisite to enable a certain degree of automatism and to transfer the semantic meaning of a model and its elements.

In publications and presentations human readable biochemical and mathematical equations or biochemical network graphs are the most convenient ways to represent models in systems biology. But for the analysis, exchange and especially the integration of models in computational tools, standardized computer readable formats are a basic requirement. Over the past years different XML-based formats have been developed (e.g. SBML [11], Biopax [12], CellML [13]) to represent models in various application areas and modeling tasks. SBML has evolved as the most widely used format to represent kinetic models. To date, more than 250 software tools support this model format [14]. Furthermore, many model repositories have been build up in recent years of systems biology research. Arguably the most popular example is the BioModels Database [1], which contains an impressive number of models (as of 2015 for example more than 500 curated models [15]). Another example is JWS Online - Model Database [3] which provides the opportunity to simulate models online. To assign biological information to the model elements (i.e. compartments, species, reactions and parameters) annotation standards have been developed. For SBML models the MIRIAM standard [16, 17] describes how semantic information can be related to the elements. The mentioned standards for model formats, model annotation and model repositories are intensively investigated research fields in systems biology [18, 19] and can support the process of structural model integration.

### Existing software supports model merging

Few scientific publications concerning the merging of network models and appropriate software tools appeared in recent years [19–21]. In general, universal xml-tools (xmldiff/patch [22]) can be used to compare and merge the xml structure of two models. But as these tools rely only on the plain xml structure there is no support for model annotation. Hence, identical elements can not be discovered based on semantic information assigned to the elements in form of annotations. The most sophisticated tool which supports a semi-automatic merging of network models of two quantitative models is semanticSBML [9]. Besides semanticSBML other software tools support structural model integration, for example, the Model Composition Tool [7] and the software PInt [23]. As in semanticSBML elements are matched based on annotations. Another software tool, for models encoded in SBML is SBMLCompose [8]. The graph merging approach supported by this tool is based on the XML code and doesn’t incorporate information which is encoded in the annotation of model elements. Also the software COPASI supports model integration to a certain degree [24]. The software Cytosolve [25] follows the idea to dynamically integrate the computations of smaller models that can run in parallel across different machines. The source code of the individual models is kept intact. Similarly the approach of Randhawa et al. [20] supports different processes of model merging. Finally, the approach followed in the modular modeling tool ProMoT [26, 27] can also provide assistance in model integration. There models can be defined as modules with interfaces which can be connected to obtain combined models. In a similar way SBML Level 3 may be used for model integration (cf. [28]). This modular language is subdivided into a core and additional packages comprising special features. The hierarchical composition package targets model integration. In the approach followed in the development of this package models are subdivided into submodels which are connected via ports.

Obviously, structural integration of models has been approached in recent years. But all aforementioned approaches and software tools only support model merging and hence structural integration. Neither considers the adaption of parameters after the merging step and specialized methods and software tools which can support this step do not exist.

## Results and discussion

In the following sections we will provide our approach to model integration. First we will introduce a workflow which subdivides the general integration task into three major steps. We then discuss challenges in structural as well as in behavioral model integration and present possible solution strategies. To illustrate challenges and solutions arising in behavioral integration, two models describing EGF and NGF signaling originally presented in [10] are integrated.

### A semi-automatic workflow based on existing standards and software

**Fig. 1:**
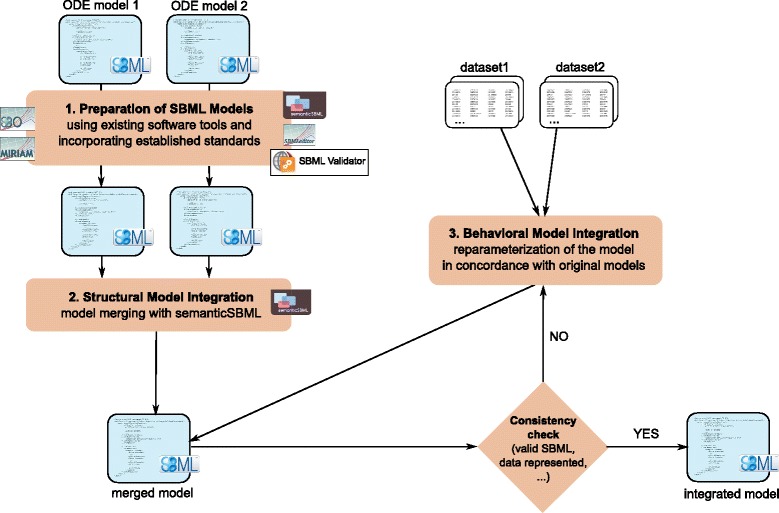
A semi-automatic workflow for horizontal model integration in three steps. The first step is the ‘Preparation of SBML Models’. The second step is the ‘Structural Model Integration’ of two SBML models that results in a merged model. This is to be checked for consistency with experimental data and eventually reparameterized in the ‘Behavioral Model Integration’ step. Several iterations of consistency check and reparameterization may be necessary to obtain an integrated model that can explain all experimental data. For a detailed description of the three steps see the main text

As pointed out in the previous section, there exists a variety of standards and tools that support structural model integration. We will present a semi-automatic workflow which incorporates many of these. This workflow consists of three major steps: ‘Model Preparation’, ‘Model Merging’ and ‘Model Reparameterization’ (see Fig. 1 for an outline of the workflow).

#### Model preparation

Prior to the merging of network models in SBML the models have to be prepared appropriately. Thereby the first task is to ensure that the units used in both models match. This might require a conversion step, where the units used in one model are converted to match those of the second model.

The goal of the model preparation step is to facilitate a unique identification of model elements. Here the software semanticSBML provides convenient features, as it allows, for example, to search a large collection of Databases for suitable annotations using keywords. Furthermore we recommend to use the SBML Validator [29] to ensure that the model is in valid SBML. In the section ‘Challenges in structural model integration’ we will point out that established annotation standards like SBO and MIRIAM are often not sufficient to discover identical model elements when signal transduction models are considered. This requires an appropriate naming scheme in combination with annotations (see Additional file 1). The names of model elements can comfortably be edited with the SBMLeditor [30]. The outcome of this step are two well prepared models in SBML format.

#### Model merging (structural model integration)

We recommend to use the software semanticSBML. In semanticSBML an initial matching of model elements can be calculated automatically. To this end information about the model elements is required to identify the overlap of two models automatically. This information has to be assigned in form of annotations and names in the prior model preparation step. The initial matching is calculated solely based on the annotations. A manual post-editing of this matching is supported by the software. Here the element names can be incorporated to solve conflicts, clear wrong matches or add matches which have not been found automatically. The outcome of this step is a new model with a fixed network structure.

#### Model reparameterization (behavioral model integration)

After the merging step the obtained model has to be tested if it is in valid SBML and if it is consistent with the experimental data (consistency check). If the merged model is not consistent with the experimental data the parameters have to be adapted. It might be necessary to pass through the reparameterization and consistency check of the model repeatedly.

### Challenges in structural model integration

When integrating two models, whether semi-automatic or by hand, the overlap of the models has to be recognized and handled, that is, identical elements have to be identified and combined in an appropriate way to obtain the merged model. In this section we describe potential complications and, where available, comment on how to resolve these.

#### Modification on different sites

**Fig. 2:**
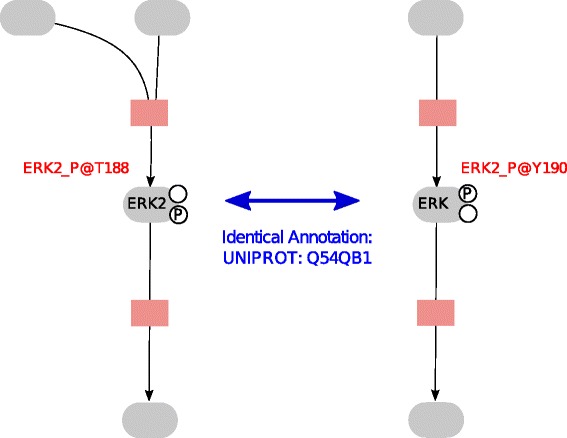
Two models describe the phosphorylated protein ERK. The recognition that ERK and ERK2 describe the same molecule can occur based on annotations. To distinguish that ERKp and ERK2p are modified on different sites (in the model shown left the threonine residue 188 is phosphorylated; in the model shown right the tyrosine residue 190 is phosphorylated) names have to be incorporated

In the MIRIAM guidelines it is defined how models and model elements can be related to entries of various databases like UniProt [31], Kegg [32], Gene Ontology [33] or ChEBI [34] using the Resource Description Framework (RDF). Following the MIRIAM standard may be sufficient to discover identical elements in metabolic models, because in general, every model component can be related to web resources. Whereas for signaling systems the annotation may not be sufficient to uniquely identify elements, as often only basic forms of molecules are available in databases. Problems will then arise because in signal transduction species often describe molecules with multiple modifications or complexes composed of several molecules with various stoichiometry. These species cannot be identified using the database annotation alone. Moreover, sometimes elements can not be found in databases. In Fig. 2 an example is shown.

Here a solution is to encode additional information in the names of model elements, for example information on modification sites and the stoichiometry in complexes. To ensure a unique identification of the model elements a naming scheme can be used. In Additional file 1 we provide guidelines how a combination of rdf annotations, SBO annotations [35] and names, following a naming scheme can be used to ensure a unique identification of model elements for signaling. These guidelines have been developed within the framework of the Virtual Liver [36].

A modeler may get the impression that annotating models and following common standards is connected with a high work load. There’s no denying, but the effort put in the annotation of models prior to structural integration is definitely not in vain. Standardized formats, annotations and curated model repositories are a general trend in systems biology to make models available and more reusable for other modelers. This trend is reinforced by many journals where models have to be uploaded in repositories in a standardized format. And, in the context of this work, if the models are well prepared, software tools can be used to perform the structural integration in a semi-automatic manner.

#### Different level of detail in reactions

**Fig. 3:**
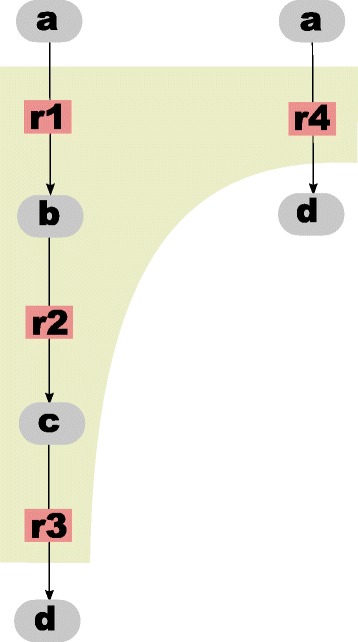
Reactions may be modeled on different level of detail. As an example the sequence of reactions on the left (r1, r2, r3) describe the same overall reaction like r4

Another challenging task is the identification of the same overall reactions which are modeled on a different level of detail (see Fig. 3). This is a task which can currently not be automatized. If the model is well prepared and the elements are annotated and named as proposed in our guidelines (see Additional file 1) the reactant and product species of the overall reaction can be recognized as equal. In many cases a graphical visualization of the model may also be helpful. The decision whether the integrated model should contain the detailed or the lumped description mainly depends on the goal of the integration task.

#### Differently modeled reactions

**Fig. 4:**
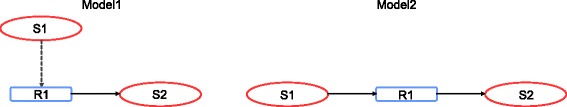
Two ways to model the creation of S2 under the control of S1: S1 acts as a modifier in model1 and as a reactant in model2

A reaction may be represented differently in different models. Both models might, for example, contain the production of *S*2 controlled by *S*1. But in one model *S*1 acts as a modifier, while in the other model *S*1 acts as a reactant (see Fig. 4). A similar situation arises, when both models contain the reaction from *S*1 to *S*2 but use different kinetic laws. In this case a decision has to be made which of the two reactions should be chosen.

#### Molecules in different compartments

If the models contain the same molecule but in different compartments a review of the compartment names and annotations should be the first step. Depending on the integration goal, an adoption of both species and an additional transport reaction between the compartments may be a solution.

#### Molecules in different states

Frequently two models contain a molecule in different states, e.g. one model contains a molecule only in an unmodified state; the other model contains the molecule only in a modified state. In most cases an adoption of both molecules and an additional modification or complexation reaction is a solution.

### Challenges in behavioral model integration

The outcome of the structural integration step is a merged model, that is, a combined model containing the elements of both models. During this structural integration parameter values (reaction rate constants and initial values) are assigned to the appropriate model elements.

The aim of behavioral model integration is to obtain a parameterized integrated model that is consistent with experimental data of the original models. As we will demonstrate below, this will usually not be the case, if all parameter values of the original models are re-used in the integrate model. Rather, parameter values assigned to model elements will have to be adjusted.

One reason is the inherent ambiguity in assigning parameters to model elements. While the choice of parameter values is easy for non-overlapping model parts where only one parameterization exists, it can be challenging for reactions in the overlap, where it is not a priori clear which parameterization is suited best. Choosing either one will almost certainly affect the ability of the integrated model to explain experimental data.

**Fig. 5:**
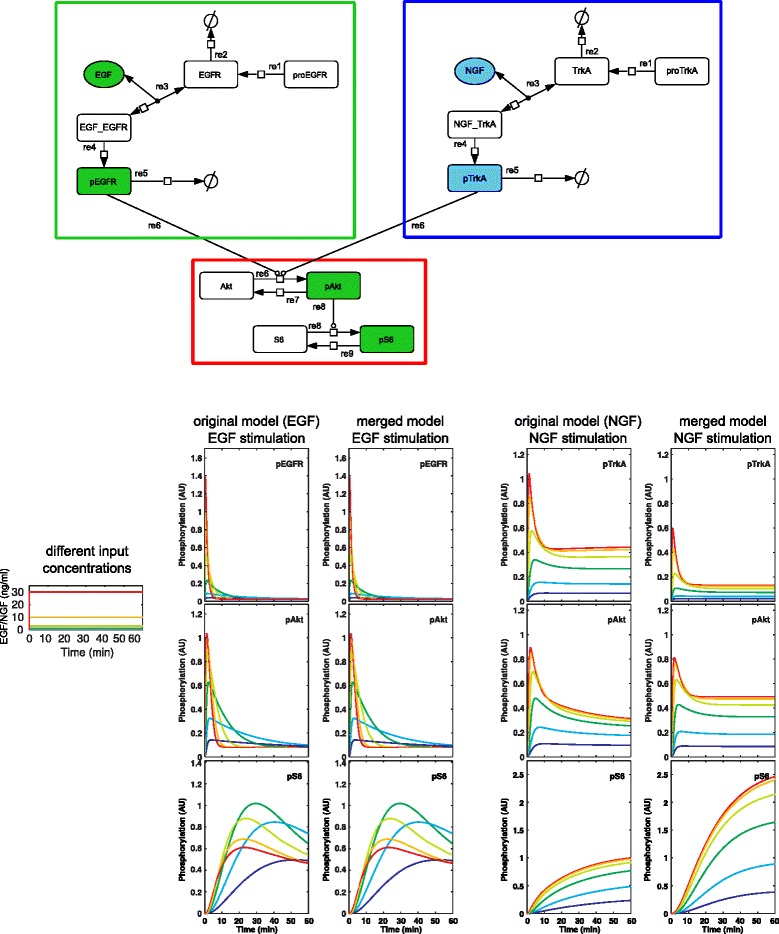
Integration example. *Upper part:* model obtained by integrating two models described in [10] (available in the BioModels Database as *B* *I* *O* *M* *D*0000000262 and *B* *I* *O* *M* *D*0000000263; the diagram is adopted from [10]). *Green box:* elements coming from EGF model alone; *blue box:* elements coming from NGF model alone; *red box:* elements coming from both models (overlap). For the non-overlapping model parts parameter values and initial concentrations are taken from the original models. For the overlap parameter values and initial concentrations of the EGF model have been chosen. *Lower part:* Simulation results. Simulations describe stimulation with different EGF and NGF concentrations, colors represent input concentrations *(left)*. *First column:* simulation of the EGF model, *second column:* the same outputs obtained from the integrated model. As expected EGF model and integrated model yield very similar simulation results. *Third column:* simulation of the NGF model, *fourth column:* the same outputs obtained from the integrated model. Here simulation results differ wildly

To illustrate this, two models describing EGF and NGF signaling have been merged. These models originate in [10], details are given in the ‘Methods’ section. For reactions that occur only in one of the models (green and blue box in Fig. 5), only one parameter set is available. But for reactions in the overlap (red box in Fig. 5) two possible parameter sets exist. For demonstration purposes the parameter values of the EGF model have been chosen for the reactions in the overlap. Consequentially, simulation results of the original EGF model can be reproduced, simulation results of the original NGF model can not be reproduced (see lower part of Fig. 5). Hence we argue that the model is not consistent with the experimental data of the original NGF model. (Whereby we assume that the original models have been consistent with experimental data. Hence, if the integrated model is able to reproduce the time courses it is also consistent with the corresponding experimental data).

Generally speaking, whenever two models A and B are integrated, choosing parameter values of model A for the overlap is expected to result in simulation results similar to those of model A; likewise, choosing parameter values of model B for the overlap is expected to result in simulation results similar to those of model B. Thus, in general, parametrizing the model overlap with values belonging to one of the models is expected to result in an integrated model that is not able to reproduce the simulation results of both original models and hence is not consistent with the experimental data of at least one model.

#### Consistency conditions

We propose to judge consistency with experimental data by means of input/output relations: whenever an input is applied a model produces a corresponding output.

**Fig. 6:**
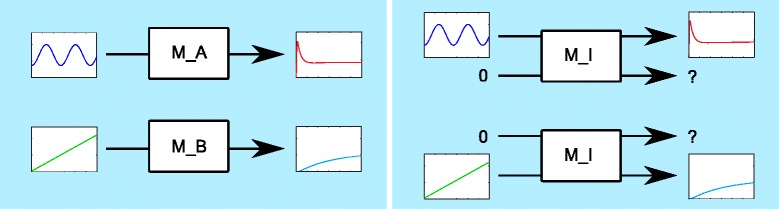
Consistency condition. *Left panel:* signal *ů*_*A*_ (blue curve) as an input to model M_A yields output *v* ¨_*A*_ (red curve), signal *ů*_*B*_ (green curve) as an input to model M_B yields output *v* ¨_*B*_ (turquoise curve). *Right panel:* the integrated model is consistent, if input (*ů*_*A*_,0) yields output similar to (*v* ¨_*A*_,?) and input (0,*ů*_*B*_) yields output similar (?,*v* ¨_*B*_)

Informally speaking, if a signal is presented to the inputs of the integrated model that come from model A, while the inputs coming from model B are set to zero, then those output signals of the integrated model coming from A should be ‘similar’ to the output of A for the same input signal (cf. Fig. 6).

To be more precise, let *u*_*A*_ denote the inputs of the merged model which originate from model A, *ů*_*A*_ the inputs of model A, *u*_*B*_ the inputs of the merged model which originate from model B, and *ů*_*B*_ the inputs of model B. Likewise, let *v*_*A*_ denote the outputs of the merged model which originate from model A, *v* ¨_*A*_ the outputs of model A, *v*_*B*_ the outputs of the merged model which originate from model B and *v* ¨_*B*_ the outputs of model B. Finally, let *u*=(*u*_*A*_,*u*_*B*_) and *v*=(*v*_*A*_,*v*_*B*_) denote input and output of the integrated model, where identical inputs and outputs are listed only once. Then *u*=(*u*_*A*_,0) denotes a signal where all inputs that originate from model A receive a signal while all those belonging only to B are set to zero and *u*=(0,*u*_*B*_) denotes a signal where the roles of A and B have been exchanged. Similarly, *v*=(*v*_*A*_,?) denotes a signal where all outputs originating from A show a specific value, while those belonging only to B may take any value and *v*=(?,*v*_*B*_) denotes a signal where the roles of A and B have been exchanged.

We say an integrated model is consistent with experimental data, if the following relations hold for all signal pairs *ů*_*A*_, *v* ¨_*A*_ used to parametrize model A and for all signal pairs *ů*_*B*_, *v* ¨_*B*_ used to parametrize model B (cf. Fig. 6):

1. Inputs *u*=(*ů*_*A*_,0) yield output *v*≈(*v* ¨_*A*_,?)
2. Inputs *u*=(0,*ů*_*B*_) yield output *v*≈(?,*v* ¨_*B*_)

To assess the similarity of the output curves we suggest to use the *χ*^2^ merit function which is often optimized in parameter estimation (see, e.g., the software PottersWheel [37]):

(1) $$ \chi^{2}(p) = \sum_{i=1}^{N} \left(\frac{y_{i}-y(t_{i};p)}{\sigma_{i}}\right)^{2}  $$

In the above formula *y*_*i*_ is the data point *i* with the standard deviation *σ*_*i*_ and *y*(*t*_*i*_;*p*) is the model value at time point *i* for parameter values *p* (see, for example, [37]).

In the following we suggest to compute two *χ*^2^-values to assess the similarity of the output of the integrated model to that of models A and B: ${\chi ^{2}_{A}}/N_{A}$ and ${\chi ^{2}_{B}}/N_{B}$. Thereby *N*_*A*_ and *N*_*B*_ are the number of data points of the respective model, *y*_*A*_(*t*_*i*_;*p*_*A*_) and *y*_*B*_(*t*_*i*_;*p*_*B*_) denote the value of the output signals of A and B at time point *i* and *y*_*I*_(*t*_*i*_;*p*_*I*_) the value of the output signal of the integrated model at time point *i*. The values ${\chi ^{2}_{A}}/N_{A}$ and ${\chi ^{2}_{B}}/N_{B}$ are then calculated as follows:

(2) $$ \frac{{\chi^{2}_{A}}}{N_{A}} = \sum_{i=1}^{N_{A}} \left(\frac{y_{A}(t_{i};p_{A})-y_{I}(t_{i};p_{I})}{\sigma_{i}} \right)  $$

(3) $$ \frac{{\chi^{2}_{B}}}{N_{B}} = \sum_{i=1}^{N_{B}} \left(\frac{y_{B}(t_{i};p_{B})-y_{I}(t_{i};p_{I})}{\sigma_{i}} \right)  $$

Now we say the integrated model is consistent, if

1. ${\chi ^{2}_{A}}/N_{A} < 1$ and
2. ${\chi ^{2}_{B}}/N_{B} < 1$.

With this consistency condition it is possible to formulate integration goals. It is often not required to obtain consistency of the integrated model with both original models equally well. An exemplary integration goal could be:

- Reproduce simulation results of model A almost exactly and reproduce simulation results of model B as well as possible, that is, obtain a parameterization such that the integrated model satisfies the condition
  (4) $$ {\chi^{2}_{A}}/N_{A} <1, {\chi^{2}_{B}}/N_{B} \approx 1.  $$

For the integration example in Fig. 5 we define the following integration goal: reproduce the time courses of the EGF model almost exactly and those of the NGF model as well as possible (i.e. $\chi ^{2}_{EGF}/N_{EGF} < 1$ and $\chi ^{2}_{NGF}/N_{NGF}\approx 1$). This integration goal guided our choice of parameter values for the elements of the overlap: we assigned the parameter values of the EGF model to the overlap.

Generally speaking, setting an integration goal can guide the initial choice of parameter values for the overlap. If the goal is to reproduce the simulation results of one of the original models almost exactly, the parameter values of this original model should be chosen for the overlap. For the non-overlapping model parts parameter values of the original models can be chosen for the integrated model. In this sense an integration goal influences structural integration.

Note that to compare the output signals of integrated and original model one may either use experimental or simulation data. To check consistency of the model presented in Fig. 5 we make use of the simulation data of the original models. For this purpose we interpret model values *y*_*A*_(*t*_*i*_;*p*) as synthetic data points *y*_*A*;*i*_ and assume normally distributed errors for these data points (10 % relative and 5 % of the maximum as the absolute error). This approach works on the assumption that the original models have been consistent with the experimental data (as is the case for the example models). If the experimental data which has been used to parametrize the original models is available an alternative approach is to use these data to judge the merged model instead of utilizing the approach with synthetic data points.

For the integration example shown in Fig. 5 we obtain $\chi ^{2}_{EGF}/N_{EGF} = 0.0004$ and $\chi ^{2}_{NGF}/N_{NGF} = 1689.3$). Hence the model is not consistent with experimental data.

#### Parameter re-fitting

To achieve a consistent model, parameter values have to be modified. Thereby the identification of those parameters that have to be re-fitted is a crucial question that is influenced by the integration goal and the position of the overlap of the two original models.

However, no general guidelines can be formulated for the identification of those parameters that have to be re-fitted. Instead a detailed understanding of merged model and integration goal are essential. In our example the aim is to preserve the time courses of the outputs of the EGF model almost exactly. Hence we select those parameters of the NGF model that do not belong to the overlap (blue box in Fig. 5).

Contrary to the structural integration there is a lack of tools and software to support the whole process of reparameterization. Software tools for parameter fitting like PottersWheel [38] or the Systems Biology Toolbox 2 (SBTOOLBOX2) for MATLAB [39] can be used instead.

**Fig. 7:**
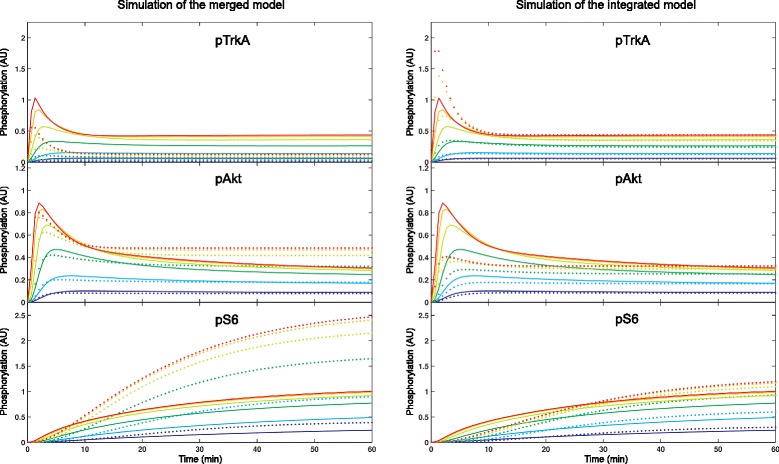
Simulation of the integrated model after merging and reparameterization

In Fig. 7 the simulation results after the merging step (left column) and after the reparameterization step (right column) are depicted for the integration example. Solid lines represent the original NGF model; dotted lines the integrated model after the respective step of the workflow. The time courses of the three output states pTrkA, pAkt and pS6 are shown after stimulation with different concentrations of NGF (colors correspond to the different NGF-stimuli used in Fig. 5). The early response can not be reproduced very well, even after reparameterization. Nonetheless, steady steady values almost coincide after the reparameterization step. Comparing entire output signals via the $\frac {\chi ^{2}}{N}$ values reveals the similarity of the signals: $\frac {\chi ^{2}_{EGF}}{N} = 0.289$ and $\frac {\chi ^{2}_{NGF}}{N} = 0.899$. Hence the integration goal is achieved. Moreover, the model is consistent according to our definition. Especially for the output pS6 the time courses can be reproduced almost exactly after reparameterization. From our point of view this output is more important than the other two because it forms the end of the signaling cascade.

## Methods

### Examining an integration example

To illustrate the important tasks in model integration which have been described in the previous sections we chose two models of signaling pathways in pc12 cells. The models of epidermal growth factor (EGF)-dependent Akt pathway and nerve growth factor (NGF)-dependent Akt pathway have been set up by Fujita et al. [10] and are publicly available in the curated branch of the BioModels Database [1] as *BIOMD*0000000262 and *BIOMD*0000000263. Each of the two models comprises 11 reactions and 11 species. With these two models Fujita et al. studied how temporal patterns in the upstream signals are transmitted to the downstream effectors. Experiments showed a decoupling of the peak amplitudes which could be reproduced with the two simple pathway models sufficiently. Frequency response analysis has been used by Fujita et al. to uncover low-pass filter properties of the three-component Akt pathways.

**Fig. 8:**
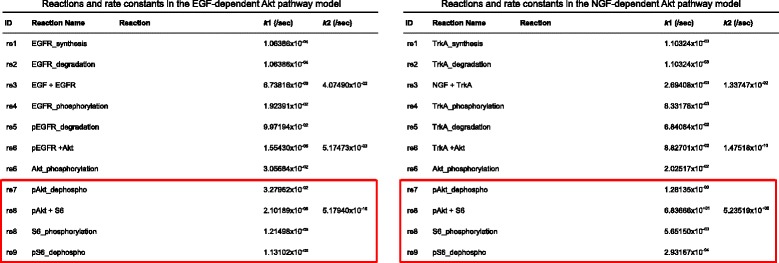
Reactions of the two BioModels *B* *I* *O* *M* *D*0000000262 and *B* *I* *O* *M* *D*0000000263 (taken from Fujita et al. [10]). The model overlap consists of the last reactions *(red boxes)* and the outputs

The overlap consists of four reactions, five species and two output states. In Fig. 8 the reactions of the original models are listed. The last four reactions of both models are equal, they solely differ in the parameters (red boxes in Fig. 8).

**Fig. 9:**
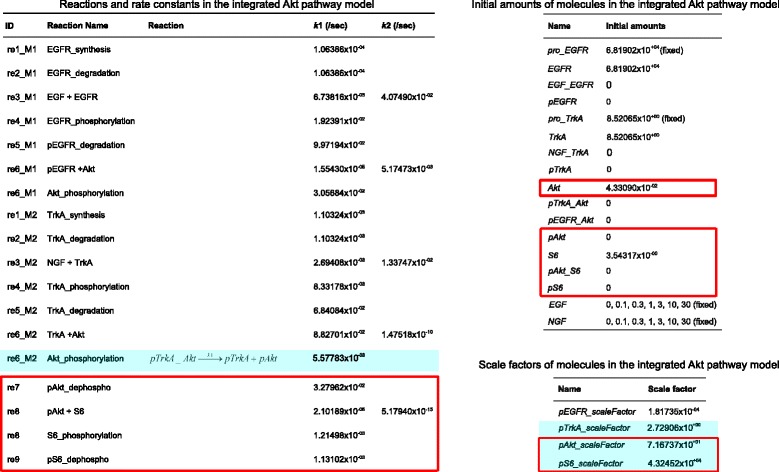
Reactions, initial amounts and scaling factors of the integrated model: parameter values of the original EGF model have been used for the overlap *(red boxes)*. Reaction parameters and scaling factors highlighted by *blue boxes* have been re-estimated

Following our model integration workflow the structural integration of the two models in SBML format is straightforward. First some annotations have been corrected, SBO annotations have been added and appropriate names have been assigned to the model elements. The annotations have been edited using the software semanticSBML, names have been edited with the software SBMLeditor. Then semanticSBML has been used to obtain a merged model. The parameterization of the EGF model was chosen for the overlap. In Fig. 5 the network of the merged model is shown. The merged model contains the EGF and NGF input species. Both signals are transmitted to the downstream effectors pAkt and pS6. Figure 9 depicts the reactions, initial amounts and scaling factors of the integrated model. Elements which have been part of the overlap are highlighted by red boxes.

As described in the ‘Results and discussion’ section difficulties arise in the selection and adaption of parameters to obtain consistency of the original models and the merged model. We will now demonstrate that the selection and adaption of parameters mainly depends on the integration goal. For the integration example the following goals can be defined:

- Preserve time courses of EGF model exactly and obtain consistency of the integrated model with the NGF model $\left (\frac {\chi ^{2}_{EGF}}{N_{EGF}} < 1, \frac {\chi ^{2}_{NGF}}{N_{NGF}}\approx 1\right)$.
- Preserve time courses of NGF model exactly and obtain consistency of the integrated model with the EGF model $\left (\frac {\chi ^{2}_{EGF}}{N_{EGF}} \approx 1, \frac {\chi ^{2}_{NGF}}{N_{NGF}}<1\right)$.
- Preserve consistency of the integrated model with the two original models $\left (\frac {\chi ^{2}_{EGF}}{N_{EGF}} < 1, \frac {\chi ^{2}_{NGF}}{N_{NGF}} < 1\right)$.

For demonstration purposes we have chosen goal one. The consequence is that we choose the model variant which contains the parameter set of the EGF model for the reactions in the overlap. After structural integration the time courses of the following outputs have been preserved exactly: pEGFR, pAKT and pS6 after stimulation with EGF and pTrkA after stimulation with NGF. The time courses of the outputs pAKT and pS6 after stimulation with NGF differ from the ones in the original model (see Fig. 5). Hence, the integrated model is already consistent with the EGF model. The challenge is now to find the parameters that are modified in a re-fitting step to obtain consistency of the integrated model with the NGF model. This will be described later in this section.

Prior to the model fitting step synthetic data points have been produced by simulating the two original models. The original data hasn’t been available. The multiple fitting functionality of the PottersWheel software [37] has been used. First we tried to fit the merged model only with data sets produced by simulation of the NGF model. Therefore we utilized six data sets with NGF step stimulation in different concentrations (as described in [10]). As an initial try four parameters (three reaction parameters of re6 (see Fig. 8) and one scaling parameter for the output pTrkA) have been fitted to reproduce the time courses of the three outputs of the NGF model (pTrkA, pAkt and pS6). This approach was not successful $\left (\frac {\chi ^{2}}{N} = 2.291\right)$.

As we want to keep all parameter values of the EGF model, the following parameters are candidates for the fitting step: the reaction parameters in the upper model branch of the NGF model, the corresponding initial concentrations and the scaling factor for the output pTrkA. If the reaction parameters and initial concentrations in the overlap or upper model branch of the EGF model or the scaling factors for the outputs pAkt or pS6 are fitted, the time courses of the original EGF model can not be reproduced exactly.

Of these, the parameters of the Akt phosphorylation reaction in the original NGF model and three scaling factors turned out to yield the best results (these parameters are highlighted in blue in Fig. 9). Because the scaling factors have an influence on the outputs in the overlap additional six data sets with EGF step stimulation in different concentrations have been utilized for the fitting. The twelve data sets each contain four time courses describing the four outputs of the integrated model (pEGFR, pTrkA, pAkt and pS6). With this approach $\frac {\chi ^{2}}{N} = 0.594$ can be achieved for all data sets, the individual quotients are $\frac {\chi ^{2}_{EGF}}{N_{EGF}} = 0.289$ and $\frac {\chi ^{2}_{NGF}}{N_{NGF}} = 0.899$. Hence both consistency conditions are fulfilled.

## Conclusions

The present work describes a semi-automatic model integration workflow. This workflow is subdivided into three major steps, model preparation, structural integration, and behavioral integration. As the first two steps are mainly concerned with the semantic meaning of model elements, one may think of these steps as ‘biological integration’. The described methods are tailored to signal transduction models. For models describing metabolism the steps can be applied similarly, but more straightforward, because the identification of model elements is less complicated. The third step, behavioral integration, focuses mainly on the mathematical aspects of the integration task. Hence, it can be considered as ‘mathematical integration’. This step and the discussed ideas can readily be applied to models describing either signaling or metabolism. Our workflow can incorporate many existing standards and software tools.

We want to emphasize that model integration is more than model merging: one has to ensure that the integrated model is consistent with the experimental data of both of the original models. Hence the choice of parameter values for the integrated model is crucial. And there is an ambiguity in assigning parameter values to the model overlap, as it is a priori not obvious which parameter values to choose (those coming from model A or those coming from model B). To guide this choice and to judge the success of the integration process we propose to formulate an integration goal. In particular, we suggest to use the $\frac {\chi ^{2}}{N}$ value for this purpose.

In most cases the integration goal will not be achievable using the parameter values of the original models. Instead at least some of the parameter values will have to be re-estimated. Thereby the identification goal may help to define suitable subsets of the parameters. The values of the corresponding parameters have then to be re-estimated given the measurement information corresponding to both models (or synthetic data obtained by simulating the original models).

Besides the $\frac {\chi ^{2}}{N}$ value other quantities may be used to formulate integration goals, for example, steady state values. Fujita et al. studied the low-pass filter properties of the two Akt pathways. Thereby so-called ‘cut off frequencies’ play an important role. These also offer an alternative way to formulate integration goals, at least for the special systems studied in [10]. Our choice of the $\frac {\chi ^{2}}{N}$ values was motivated by the following ideas: steady state values do not contain information about the time courses and ‘cut off frequencies’ are a specific property of the system studied in [10] and hence may not be generalized easily.

## Additional file

Additional file 1
**Requirements in model preparation (naming scheme and annotation).** (PDF 148 kb)
