# Supplementary material for: Challenges in horizontal model integration
Source: BMC Syst Biol. 2016 Mar 11;10:28. doi: 10.1186/s12918-016-0266-3 (PMC4788958; doi:10.1186/s12918-016-0266-3)
Supplement: Additional file 1 — Requirements in model preparation (naming scheme and annotation). (PDF 148 kb) [file 12918_2016_266_MOESM1_ESM.pdf]

Requirements for Integration of Signal-Transduction Models

Katrin Kolczyk, Carsten Conradi

February 19, 2015

1 Introduction

We focus on a model integration process where established quantitative models of signal transduction set up by different groups should be combined to obtain a larger model. To avoid redundancy in the integrated model it is important to detect overlapping parts. Therefore model elements have to be identifiable to decide which elements of the different models describe the same biological objects. This document provides a guideline on how to prepare mathematical models of biological systems encoded in the Systems Biology Markup Language (SBML, [1], [2]). We prospose to use the software semanticSBML [3, 4] to perform the integration of network models. All models intended for integration with other models should be consistent with the listed requirements and recommendations.

2 Requirements for Models

In order to detect overlapping parts, models prepared for integration have to fulfill all the requirements listed below. Explanations how to achieve these requirements are given in gray.

a) The two models have to be encoded in SBML. Thereby level 2 and version 3 or 4 (L2V3 or L2V4) or higher has to be used. Furthermore, it is important that both models use the same level and version of SBML. This requirement is tailored to the integration with the software semanticSBML. Recently, SBML level 3 core has been released. But at the moment the software semanticSBML supports only handling of models specified in SBML level 2.

To convert your SBML model into another level and version we recommend to use either Copasi [5] or CellDesigner [6]. You can import your model in one of the tools and choose the desired level and version in export.

b) The models have to be in valid SBML.

You can check validity of your model encoded in SBML with the SBML-Validator [7]. The validation rules can be found in the appendix of the SBML Level 2 specification [2] .

c) The modeling formalism has to be the same for both models. Furthermore, models have to be deterministic and non-spatial continuous. Even though in principle models containing events can also be combined but should be avoided as they need a special handling.
The modeling formalism has to be annotated using the Systems Biology Ontology (SBO) [8]. The corresponding SBO-term for a non-spatial continuous framework is `SBO:0000293`.

d) The organism has to be the same for the two models and the taxonomy information has to be available in the model annotation (see point e)).

e) Each model prepared for annotation has to be annotated according to the MIRIAM standard [9].

The following information needs to be present in the model: model creator and contact information, creation date, last modification date, taxonomy information, biological assumptions, reference to publication or report containing detailed information on the model, i.e. describing function and expected results reproducible. These so-called model annotations can directly be edited during model setup e.g. with the modeling software Copasi. For subsequent model annotation semanticSBML provides comfortable editing functionality.

f) Instantiation of model simulations must be possible, therefore initial conditions and parameters have to be available. Furthermore kinetic expressions for all reactions have to be defined and computational cycles have to be avoided, i.e. for each quantity there is one assignment. This is also described in [10].

To instantiate simulations the software Copasi can be used.
Algebraic loops should not occur because < algebraicRule > tags are not allowed in models prepared for integration (see point i)). Loops emerging because of < assignmentRule > tags should be detected in the SBML-Validator.

g) Each species, reaction and compartment must contain at least one MIRIAM compliant annotation. Besides naming conventions guidelines for the annotation of unmodified and modified molecules, complexes, reactions and compartments are described in the next section.

The annotation of model components can also be edited with semanticSBML.

h) For integration with the recommended software semanticSBML, the models must not contain algebraic rules.

The software tool we recommend for model integration does not support algebraic rules (defined by < algebraicRule > .. < /algebraicRule > in the SBML model). You can try to change the algebraic rule into an assignment rule for one variable defined by this rule. If this is not possible, create an additional parameter and use an assignment rule for this parameter or remove the algebraic rule and add it in revised form to the integrated model. Additional parameters need to be corrected after integration as well.

i) Conservation relations have to be fulfilled.

With semanticSBML the conservation of atom numbers within a reaction can be checked. Mass conservations can be checked with Copasi.

j) semanticSBML check does not report errors for both models.

You can upload and check the models in the semanticSBML web-interface [4].

k) The same units have to be used for substances, volume, time, area and length in both models. Thereby only SI units are allowed, these are declared in the SBML specification [2]. The units for all parameters have to be quite clear and consistent.

The units of a model can comfortably be edited with Copasi. But be careful, all values have to be revised after changing the units.
Whether units for parameters are defined and consistent can either be checked with the SBML-Validator (you have to tick the box for 'Check consistency of measurement units associated with quantities' prior to validation) or Copasi ('?' is written in the unit column in the parameter overview if the units are not specified or do not match the inferred ones). The SBML-Validator also gives a detailed description of the warning and hints for the intended units. The editing of parameter units and values can be done with SBMLeditor [11].

l) Choose intuitive and clear names for the elements
(according a proposed naming scheme, see section 4).

If it is not feasible to change the names of species, put the name compliant with the naming scheme into the notes section of the respective species. For this purpose use the following prefix 'species name for model integration according naming scheme:'. The scheme compliant name has to be specified either as name for the species or in the notes section. The name field of the species is the preferred location.

m) If cofactors like ATP/ADP, NAD/NADH are incorporated explicitly in the model they have to be described by global parameters or species in SBML. If they are used as local parameters an annotation is not possible.

3 Further Recommendations

- Use unique names and controlled vocabularies for kinetic parameters and reactions, math expressions and compartments.

  The SBML elements should have assigned SBO terms if possible. The most specific one should be taken (e.g. `SBO:0000190` for a Hill coefficient).

- If semanticSBML is used (as is recommended) event tags should be removed from the model prior to integration.

  Events are currently not supported in model integration with semanticSBML. They have to be added into the integrated model in a revised form.

- Try to avoid lumped reactions and species in both models because this may restrict integration (this is also described in [10]). If needed, use them in a systematic manner or at least describe unambiguously. If annotated correctly lumped reactions can be detected as they are annotated with 'has part' in most cases. However, it may be difficult to identify two reactions as equal if they are modeled on different levels of detail. Generally, the decision if the overlap of two models is well identifiable and the level of detail matches has to be made from case to case.

- Try to use realistic non-negative values for concentrations to enable incorporation of this information in the integration process.

4 Naming and Annotation of Model Elements

This section will provide a guideline for naming and annotation of elements in SBML models. Annotations for model elements have to follow the MIRIAM standard [9], [12]. Species can be annotated with respect to databases like UniProt for Proteins, KEGG for proteins, genes, metabolites and cofactors, ChEBI for cofactors, metabolites and small molecules. In conjunction with element annotation the proposed naming scheme will facilitate model reuse and integration. Currently, there is no common naming scheme which is uniformly applied in systems biology models. The proposed naming conventions will only consider species and compartments. Identification of reactions, parameters and mathematical expressions is not appropriate by name. Nevertheless, intuitive names will make the identification much easier.

The following sections will describe how compartments, unmodified and modified molecules and complexes with various stoichiometry of parts should be annotated and named. Furthermore some general annotation guidelines will be given.

4.1 Past and Related Work

Different guidelines and standards for the naming of genes and proteins, especially for enzymes exist. A reference collection can be found on [13]. Mostly the nomenclatures are organism specific (e.g. for yeast, human or mouse) and consider only the basic forms of species (unmodified and no complexes composed of smaller identifiable parts). Besides systematic names, synonyms (often including short and common names) are provided in databases we suggest to use for the annotation of species. We propose to use the short names because systematic and common names are often too long to be feasible in models.

Some software tools facilitate species names describing modifications and composition of complexes, e.g. BioNetGen [14] and PottersWheel [15]. But if all molecules have to follow the rules, this would also lead to complicated names for simple molecules. With our naming conventions we try to support short names for simple species, the building of complex names is influenced by the BioNetGen language. Naming for modified molecules is similar to SBGN [16].

4.2 Naming and Annotation of Species Types

For a better identification of species a combination of the machine-readable annotations and the human-readable species names should be used. The

names are composed like the species themselves. The smallest unit is a *basic species* (described in 4.2.3) which is a simple molecule like a protein or a metabolite. Modified forms of these basic species are named *modified species* (see 4.2.4). A *complex* (4.2.5) is a combination of basic and/or modified species with defined stoichiometry.

4.2.1 rdf Annotation

Established databases, e.g. KEGG compound, UniProt for proteins, ChEBI for cofactors and metabolites have to be used for the annotation and naming of species. All allowed databases are listed on [17]. To prevent synonyms, the closest, most similar annotation for each basic species must be used.

In SBML each element can contain a set of machine-readable rdf annotations following the MIRIAM standard. A MIRIAM compliant rdf annotation is a relation from a SBML element (e.g. a species) to a database entry. Predefined qualifiers can be used for this relation, e.g. hasPart, hasProperty, isVersionOf and so forth. All allowed qualifiers are listed on [18].

The three species types (basic species, modified species, complexes) have to be annotated as follows:

- A basic species (unmodified molecule) has to contain exactly one annotation with the `is` qualifier.
- A modified species (basic species with covalent modification) has to contain exactly one annotation using the qualifier `isVersionOf`. Furthermore, for each type of modification one annotation with `hasPart` qualifier has to be specified. For this purpose the ChEBI or the KEGG Compound database should be used if possible (e.g. a phosphorylation can be annotated with the identifier: `C00009` from the KEGG Compound database).
- Complexes must have assigned a set of annotations with qualifiers `hasPart` for basic species and a set of annotations with qualifiers `hasVersion` for modified species contained in the complex.

  For example: For the phosphorylated receptor-ligand complex EGF.EGFR_P assign an `hasPart` annotation for the basic species EGF and an `hasVersion` annotation for the modified species EGFR_P.

Besides these annotations with the qualifiers specified above the species can contain additional annotations, e.g. `isDescribedBy` to link a species to the literature that describes the concentration of it. Furthermore if there are species that describe the same substance but are located in different

compartments, the location should be specified with an `occoursIn` qualifier. This holds for the entire species. Every species should have only one single annotation for the location. If a species can be annotated and named as modified form or as basic species, it must be expressed as basic species, for example use 'ATP' instead of 'ADP_P'. Furthermore, the primary ID from the database should be taken.

4.2.2 SBO Annotation

Besides rdf annotations SBML elements can contain annotations with SBO terms [19],[20],[21]. SBO terms can be used in SBML L2V3 and higher versions. They should be assigned to SBML elements whenever they can be applied because this may simplify decisions in the manual adjustment of the initial matching based on rdf annotations. We recommend to assign SBO terms to reactions, parameters, math expressions and compartments (e.g. a phosphorylation reaction should have assigned the SBO term `SBO:0000216`). The ontology can be browsed in: [8]

4.2.3 Basic Species

The name of a basic species should be informative for other modelers and describe the intended meaning of simple molecules. These basic species can be proteins, mRNA, genes, cofactors, metabolites or other small molecules.

Existing standards and conventions have to be applied whenever possible, e.g. for gene and protein names. If available it is recommended to use either the short name from the database entry also used for the annotation or commonly used explicit and concise names in literature. If several isoforms exist, the name has to reflect which isoform is meant. If one substance which occurs in several compartments is modeled as different species, the name of these species will be equal. For this cases we propose to precede the species name by the compartment name. This prefix has to be separated from the name by a tilde. This holds for the complete species. For example use 'cyt∼ATP' for cytosolic ATP. Abbreviations for the compartment names are given in Table 3. Furthermore, context independent names have to be used, i.e. the name should be understandable independent of model context. Thus use the protein name instead of role, e.g. 'Mapk1' instead of 'kinase' (receptor, ligand, etc.). Avoid references to the taxonomic species, e.g. human, mouse, rat, within the name. The taxonomy information has to be available in the model annotation (see Section 2). Furthermore, the names for basic species must be used consistently throughout the model, i. e. if the molecule also occurs in modified form or in complexes the basic species name must be part

of the names of these molecules.

For the basic species names the following syntax rules have to be applied:

- Only use alphabetic characters and numbers from ASCII, i.e. 'a'..'z', 'A'..'Z', '0' ..'9', and '-' in basic species names.
- No formatting (e.g. italic, bold or underlined characters), accents, superscripts or subscripts are allowed within the name field of SBML. To distinguish between a gene and a protein SBO terms or an optional prefix can be used (see below).
- Take care of case sensitivity: Capitalization has to follow the conventions for protein or gene symbols, e.g. all uppercase for proteins of human, mouse or rat. For human genes all letters must be in uppercase. For mouse and rat genes the first letter must be uppercase and the remaining letters in lowercase.
- Names should be given in English language. If different spellings exist, American spelling must be used.
- Special characters, e.g. Greek symbols must be spelled out, e.g. 'TGF-beta' or 'TGFbeta'.

Material type and conceptual type: If the SBO annotation field of the species is not needed for any other information the type of a species (e.g. protein or mRNA) has to be annotated with SBO, see Table 1. Otherwise the type can be encoded in the basic species name as prefix using controlled vocabulary. To distinguish the type from the name for the basic species a defined syntax has to be used. The prefix has to be composed of 'mt' or 'ct' for material or conceptual type, separated by ':' from the label, see Table 1. The type and the remaining basic species name are separated by '_'. Examples are 'mt:prot_ERK', 'mt:prot_TIM' or 'ct:mRNA_TIM'. The distinction between material type and conceptual type and the labels are introduced in the SBGN specifications[22].

4.2.4 Modified Species

Names for modified species contain the name of the basic species and any present modifications. For each modification the kind of modification and optionally the site which is modified has to be specified. Specification of sites allows to distinguish between species carrying the same modifications at different sites. For example ERK, phosphorylated at Y190 or T188, carry in both cases exactly one phosphorylation.

Name	Label	SBO term
Non-macromolecular ion	mt:ion	SBO:0000327
Non-macromolecular radical	mt:rad	SBO:0000328
Ribonucleic acid	mt:rna	SBO:0000250
Deoxyribonucleic acid	mt:dna	SBO:0000251
Protein complex	mt:prot	SBO:0000297
Polysaccharide	mt:psac	SBO:0000249
Gene	ct:gene	SBO:0000243
Transcription start site	ct:tss	SBO:0000329
Gene coding region	ct:coding	SBO:0000335
Gene regulatory region	ct:grr	SBO:0000369
Messenger RNA	ct:mRNA	SBO:0000278

Table 1: Predefined values for material and conceptual types

To add the information about modification a special syntax has to be applied. A modification is added to the basic species name as a suffix separated by '_'. Further modifications can also be added separated by '_'. The label characterizes which kind of modification is present. Possible labels are taken from the SBGN specification [22], see Table 2. An example for a modified species name is 'EGFR_P_P'.

Name	Label
Acetylation	Ac
Glycosylation	G
Hydroxylation	OH
Methylation	Me
Myristoylation	My
Palmytoylation	Pa
Phosphorylation	P
Prenylation	Pr
Protonation	H
Sulfation	S
Ubiquitination	Ub

Table 2: Labels for covalent modifications

Modification can also describe a conformation or state of a domain. Therefore additional labels for states or covalent modifications can be defined but need to be documented. Values for state labels may be: 'active', 'inactive', 'open', 'closed', e.g. 'ERK_active'. But labels are not allowed to

start with a number to distinguish between modifications and stoichiometry within a complex.

Modification sites: The modification site specifies the location. Each label for a modification can be followed by a site separated by '@'. This syntax is similar to 'state@variable' notation for modifications in SBGN. Only one identifier for each site must be used. Avoid synonyms for the same site, e.g. 'ERK_P@T188', 'ERK_P@T' or 'ERK_P'. Instead use only one consistently within the whole model. Recommended is 'ERK_P@T188' as it characterizes the modification best while still being reasonably short.

It is recommended for site identifiers to include domain, amino acid, nucleic acid or chemical symbol together with position in sequence. This provides context for position information and reduces ambiguity. Examples are Fructose_P@C1_P@C6 or 'ERK_P@T188'.

Names of modification sites are composed from characters and numbers from ASCII. Site identifier starting with a number, or only a number are valid, e.g. 'Fructose_P@1_P@6'.

4.2.5 Complexes

Names for complexes are composed from names of all contained modified and basic species. Names for modified species are specified as given in the previous section. Character for separation between the parts of the complex is '.' (as in the BioNetGen language). For example 'EGF.EGFR' describes the complex between EGF and EGF-receptor. The parts of a complex have to be concatenated in alphabetic order.

Modifications or different states of the whole complex can be specified as suffix for the complex enclosed in parentheses. But modifications of the complex are recommended to be expressed as modifications of the parts. For example use 'EGF.EGFR_P' instead of '(EGF.EGFR)_P'.

If the same part occurs multiple times in a complex there are two possibilities to address this using the species name. The parts can be listed multiple times or the stoichiometry can be specified as positive integer number separated by '_' from the part. For example the EGF-receptor dimer can be written as 'EGFR.EGFR' or 'EGFR_2'. A stoichiometry of one is the default and must not be given. Stoichiometry can also be given for more than one part, for example the dimer of EGF receptor with two ligands bound can be specified as '(EGF.EGFR)_2' or 'EGF_2.EGFR_2'.

4.3 Naming and Annotation of Compartments

Machine-readable rdf annotations (explained in 4.2.1) and appropriate names are also essential to identify compartments. All compartments of the model have to be annotated with the Gene Ontology (GO) [23]. The allowed annotations are listed in Table 3. The corresponding names for the compartments have to be the same as defined in GO: *membrane, cytosol, nucleus, vacuole, mitochondrion, extracellular* (a synonym for the extracellular space). Note that *cell* is not a valid compartment. If no compartment is defined, the default value is *cytosol*.

Compartment name	GO Identifier	Abbreviation
Extracellular space	GO:0005615	Ext
Membrane	GO:0001602	Mem
Cytosol	GO:0005829	Cyt
Nucleus	GO:0005634	Nuc
Vacuole	GO:0005773	Vac
Mitochondrion	GO:0005739	Mito

Table 3: Allowed annotations for compartments. Exceptionally, compartment names have to be used in the species names to ensure their uniqueness. For this cases we suggest to use the abbreviations listed in the final column.

4.4 Naming and Annotation of Reactions and Parameters

The naming and annotation of reactions should also be done as precise as possible. We propose to use a descriptive name of the process in the name of the SBML element (e.g. 'MK2_phosphorylation'). In table 4 frequent processes and recommended rdf and SBO annotations are given.

For parameters we also recommend to use precise descriptive names and the SBO annotation `SBO:0000002`.

Process type	GO Identifier	SBO
Gene Expression	GO:0010467	SBO:0000183
Transport Reaction	GO:0006810	SBO:0000185
Phosphorylation	GO:0016310	SBO:0000216
Dephosphorylation	GO:0016311	SBO:0000330
Protein Degradation	GO:0030163	SBO:0000179
RNA Degradation	GO:0006401	SBO:0000179
Protein Synthesis	GO:0006412	SBO:0000184
...		

Table 4: Annotation of frequent reactions in signaling

References

[1] Hucka M, et al.,
The systems biology markup language (SBML): a medium for representation and exchange of biochemical network models.
Bioinformatics, 9(4):524-531, (2003)

[2] Hucka M, et al.,
Systems Biology Markup Language (SBML) Level 2: Structures and Facilities for Model Definitions.
Available from Nature Precedings
`http://dx.doi.org/10.1038/npre.2008.2715.1` (2008)

[3] Krause F, Uhlendorf J, Lubitz T, Schulz M, Klipp E, Liebermeister W,
Annotation and merging of SBML models with semanticSBML,
BIOINFORMATICS, 26(3), 421-422, 2010.

[4] semanticSBML 2.0: `http://www.semanticsbml.org/`

[5] COPASI 4.6 (Build 32): `http://www.copasi.org/`

[6] CellDesigner 4.1: `http://www.celldesigner.org/`

[7] SBML-Validator (Version of 2010-10-14):
`http://sbml.org/Facilities/Validator`

[8] Systems Biology Ontology: `http://www.ebi.ac.uk/sbo/main/tree.do`

[9] Minimal Information Required In the Annotation of Models
(November 2010): `http://biomodels.net/miriam/`

[10] Liebermeister W, Validity and combination of biochemical models. *Proceedings of 3rd International ESCEC Workshop on Experimental Standard Conditions on Enzyme Characterizations*, Frankfurt: Beilstein Institute, 2008.

[11] Rodriguez N, Donizelli M, Le Novère N, SBMLeditor: effective creation of models in the Systems Biology Markup Language (SBML), *BMC Bioinformatics*, 8:79, 2007

[12] Le Novère N, et al., Minimum information requested in the annotation of biochemical models (MIRIAM). *NATURE BIOTECHNOLOGY*, 23(12), 1509-1515, 2005.

[13] Protein nomenclature publication list: `http://www.uniprot.org/docs/nomlist.txt`

[14] Faeder J R, Blinov M L, Hlavacek W S, Rule-based modeling of biochemical systems with BioNetGen. *Methods in Molecular Biology*, p 1-55, 2009

[15] Maiwald T, Timmer J, Dynamical Modeling and Multi-Experiment Fitting with PottersWheel *Bioinformatics*, 24(18), 2037-2043. 2008

[16] Le Novère N, et al., The Systems Biology Graphical Notation *Nature Biotechnology*, 27(8), 735-741, 2009

[17] Overview of the different data types stored in MIRIAM Database: `http://www.ebi.ac.uk/miriam/main/datatypes/`

[18] Biomodels qualifier: `http://biomodels.net/qualifiers/`

[19] Le Novère N, Courtot M, Laibe C, Adding semantics in kinetics models of biochemical pathways. *Proceedings of the 2nd International Symposium on experimental standard conditions of enzyme characterizations.*, 2007

[20] Le Novère N, Model storage, exchange and integration. *BMC Neuroscience*, 7(Suppl 1):S11, 2006

[21] M. Courtot et al., Controlled Vocabularies and semantics in systems biology, Molecular Systems Biology, 543(7), 1744-4292, 2011

[22] Moodie S, et al., Systems Biology Graphical Notation: Process Description language Level 1. Available from Nature Precedings http://dx.doi.org/10.1038/npre.2009.3721.1 (2009)

[23] Gene Ontology: http://www.geneontology.org/
